# Morphea Involving the Lips and Gingiva: A Rare Case Report

**DOI:** 10.7759/cureus.51202

**Published:** 2023-12-27

**Authors:** Hamad Albagieh, Rana S Alshagroud, Abdullah M Aladnan, Bader Aldosari, Yara B Alburaykan, Lama Y Almashham, Afyaa A Alqasem, Areen A Alyahya, Nada I Aboheimed

**Affiliations:** 1 Oral Medicine and Diagnostic Sciences, King Saud University, Riyadh, SAU; 2 Dermatology, Albaha Hosptal, Albaha, SAU; 3 College of Dentistry, King Saud University, Riyadh, SAU

**Keywords:** oral pathology, linear morphea, oral cavity lesion, localized scleroderma, en coup de sabre morphea

## Abstract

Morphea is a subtype of scleroderma that does not involve Raynaud's phenomenon or internal organ involvement. It is a connective tissue disease that features the excessive deposition of collagen in the dermis and subcutaneous tissue, leading to a thickening of the dermis and subcutaneous tissue, eventually forming a scar-like lesion.

We represent a 19-year-old male Saudi patient displaying a white patch on the marginal gingiva of tooth #21 and multiple yellowish papules on the outer surface of the lip. Both teeth #21 and #22 have experienced recession and bone loss. The patient’s clinical history and histopathology revealed characteristic features of localized scleroderma. A treatment was proposed involving immunosuppressants, methotrexate, and pimecrolimus cream along with topical corticosteroids and excimer laser therapy (308 nm). The patient followed the treatment plan for a full month and the white patch quickly improved for the patient. Afterward, the patient has been taking only methotrexate with a significant but gradual improvement. In this paper, we discuss the differential diagnosis to be considered and present an unusual occurrence of localized scleroderma in the oral cavity.

## Introduction

Scleroderma is a condition that affects the connective tissue in the body, primarily affecting the mucous membranes and skin, exhibiting an excessive amount of collagen deposits in the dermis and subcutaneous tissue, resulting in increased thickness of the dermis and subcutaneous tissue. This eventually forms a scar-like lesion. There are multiple types of scleroderma: morphea, which is the localized form, as well as plaque morphea, bullous morphea, linear scleroderma, and systemic sclerosis or systemic scleroderma [1]. It has been observed that both children and adults experience it at the same rate [2].

Morphea is an uncommon skin disorder, with rates of incidence ranging from 3.4 to 27 cases for every 100,000 individuals. It is prevalent to a greater extent in women than in men [3]. It is an uncommon condition impacting mesenchymal tissues. Unlike systemic scleroderma, this condition does not involve Raynaud's phenomenon, a disorder characterized by the narrowing of the blood vessels in the extremities, leading to restricted blood flow and systemic organ involvement [4,5]. The etiology of morphea is yet unknown trauma, autoimmunity, radiation, and infections such as Borrelia infection are considered to be risk factors that may trigger the onset of morphea [1,2,6,7]. While the treatment options for oral mucosal morphea are limited, current therapeutic approaches, including topical corticosteroids, immunomodulatory agents, and phototherapy, aim to alleviate symptoms and improve patient quality of life [8].

Despite the available research, more studies are needed to further elucidate the mechanisms underlying oral mucosal morphea and to develop effective therapeutic strategies. This introduction aims to provide a brief overview of the condition, drawing on the relevant literature to underscore its significance in the realm of oral health and dermatology. Here, we present an unusual occurrence of localized scleroderma in the oral cavity.

## Case presentation

**Figure 1:**
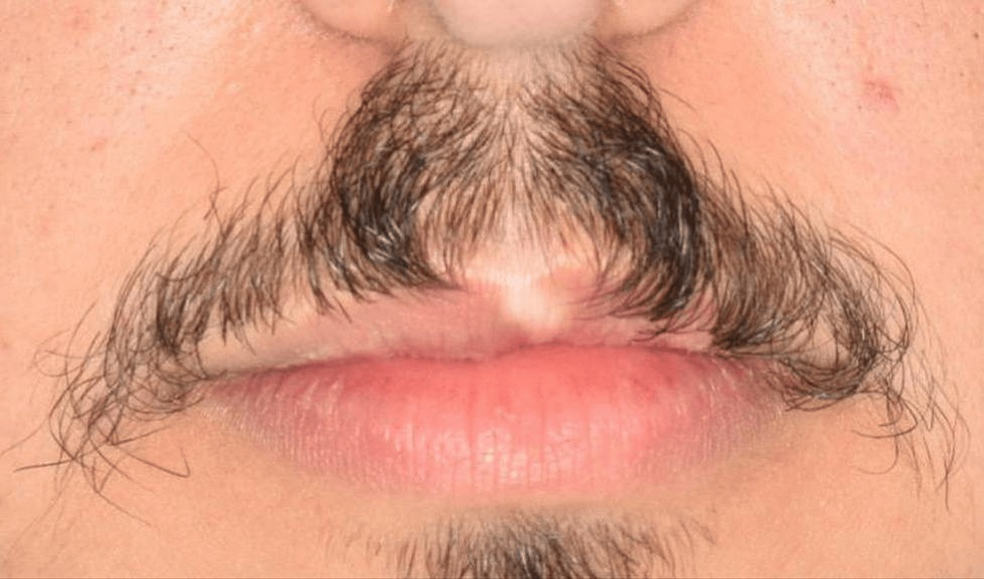
The extra-oral photograph shows a linear, white, pale, blanched appearance involving a central portion of the vermilion border of the upper lip below the philtrum

**Figure 2:**
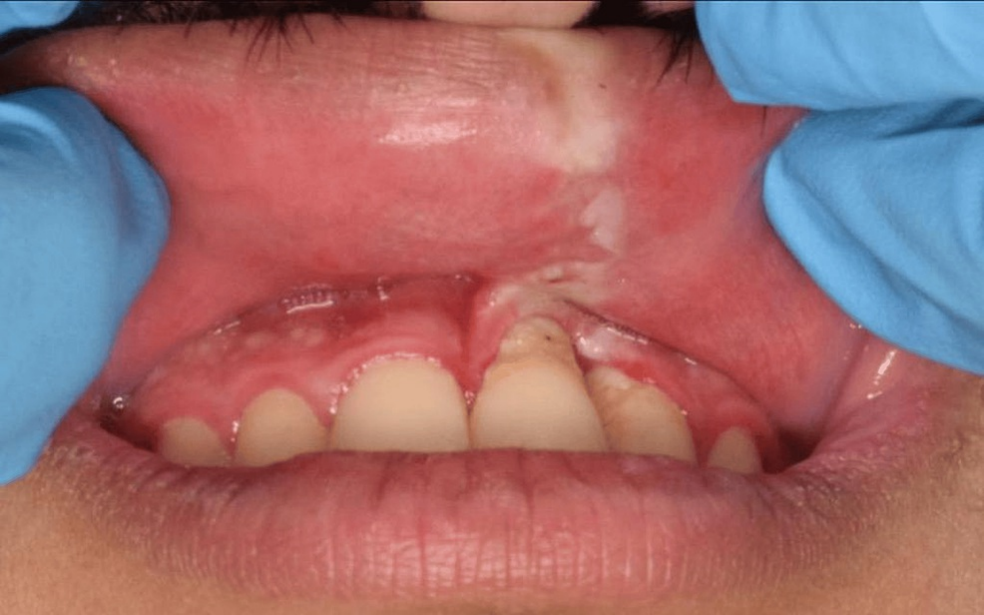
The intra-oral photograph shows a linear white patch involving the outer and inner surfaces of the upper lip, extending intra-orally into the marginal gingiva and obliteration of the mucolabial vestibule in relation to tooth #21

**Figure 3:**
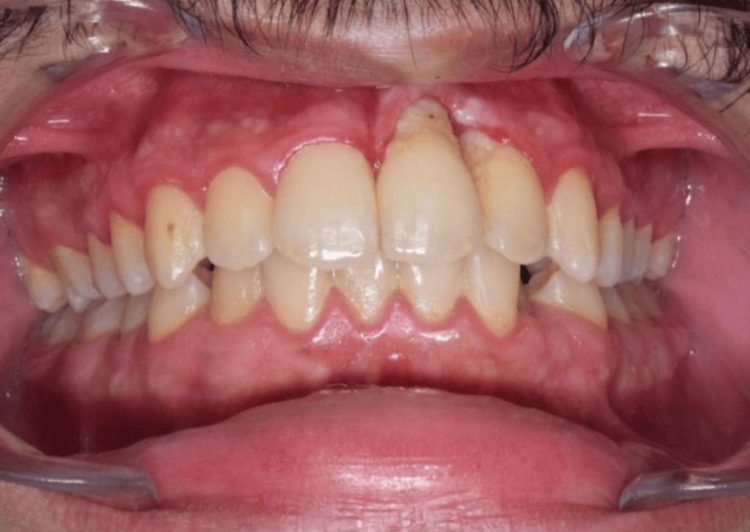
The intra-oral photograph shows that both teeth #21 and #22 experienced recession and bone loss, but it was more pronounced in tooth #21

A 19-year-old Saudi male patient, unaware of any medical conditions, non-smoker with fair oral hygiene, presented to the oral medicine clinic at King Saud University with a white patch on the marginal gingiva of tooth #21 and discrete whitish papules on the outer surface of the lip (Figure 1). The lesions had been progressively increasing in size over one year. Both teeth #21 and #22 experienced recession and bone loss (Miller's class III) but it was more pronounced in tooth #21 (Figure 2 and Figure 3). Teeth were vital but exhibited grade III mobility, a probing depth of approximately 5-6 mm was recorded around both teeth, and the patient reported sensitivity, but no pain associated with the affected teeth. The patient had previously been seen by a dermatologist, who confirmed that no other areas were involved.

**Figure 4:**
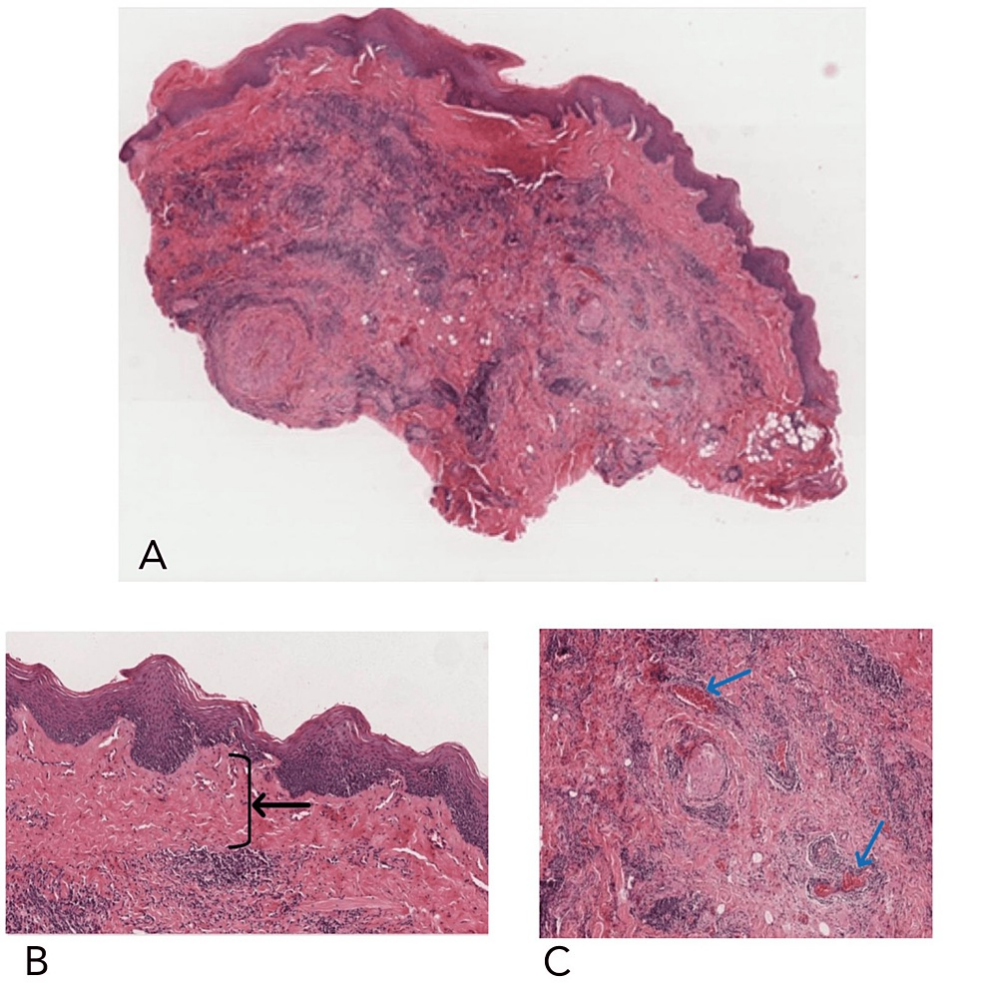
Light microscopy of the biopsy specimens from the left labial mucosa A. Mild hyperkeratotic parakeratinized stratified squamous epithelium with variable thickness in addition to fibrosis of the underlying connective tissue, (original magnification: 50x). B. Subepithelial hyalinization of hypocellular dense amorphous eosinophilic material (indicated by the black arrow) (original magnification: 100x). C. Presence of peri-vascular lymphocytic infiltrates (indicated by the blue arrows) (original magnification: 100x)

To confirm the diagnosis, two incisional biopsies were performed, one from the gingival area and the other from the labial mucosa fixed in formalin. The histopathological examination revealed characteristic features of localized scleroderma. No immunohistochemistry markers were done, but clinical presentation and histopathological findings were compatible with localized scleroderma. The specimens consisted of a wedge of oral mucosa (bisected) (Figure 4). The mucosa was lined by two types of stratified squamous epithelium, keratinized and non-keratinized with minimal subepithelial connective tissue. The non-keratinized mucosa showed basal cell melanosis, a band of inflammatory cells in the superficial lamina propria, and a vague band of hyalinization. The keratinizing mucosa exhibited thin, elongated, and reticulated rete ridges with underlying dense hypocellular collagenous tissue. There were scattered blood vessels, some of which were collapsed and a few nerve fibers were observed, These findings confirmed the diagnosis of localized scleroderma, ruling out other potential differential diagnoses such as vitiligo or lichen sclerosis.

Following the diagnosis of localized scleroderma, the patient was prescribed triamcinolone and Dermovate ointment for one month, as part of the treatment plan. Regular follow-up appointments were scheduled to monitor their response to the treatment and assess disease progression.

Treatment

**Figure 5:**
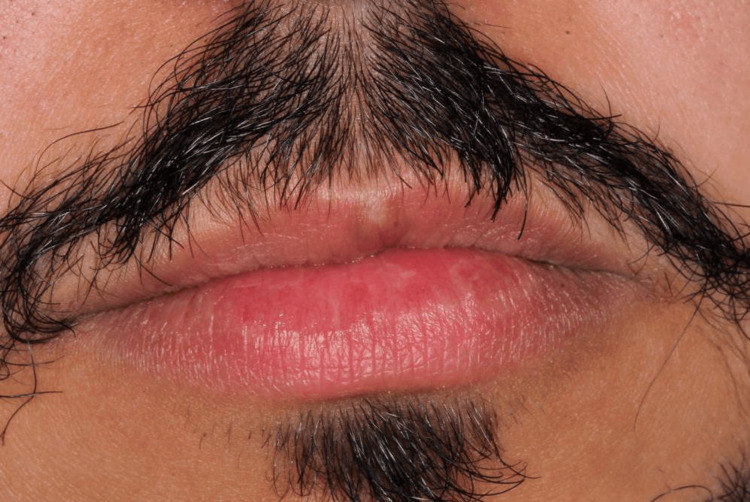
Extra-oral photograph showing a distinct improvement following treatment

**Figure 6:**
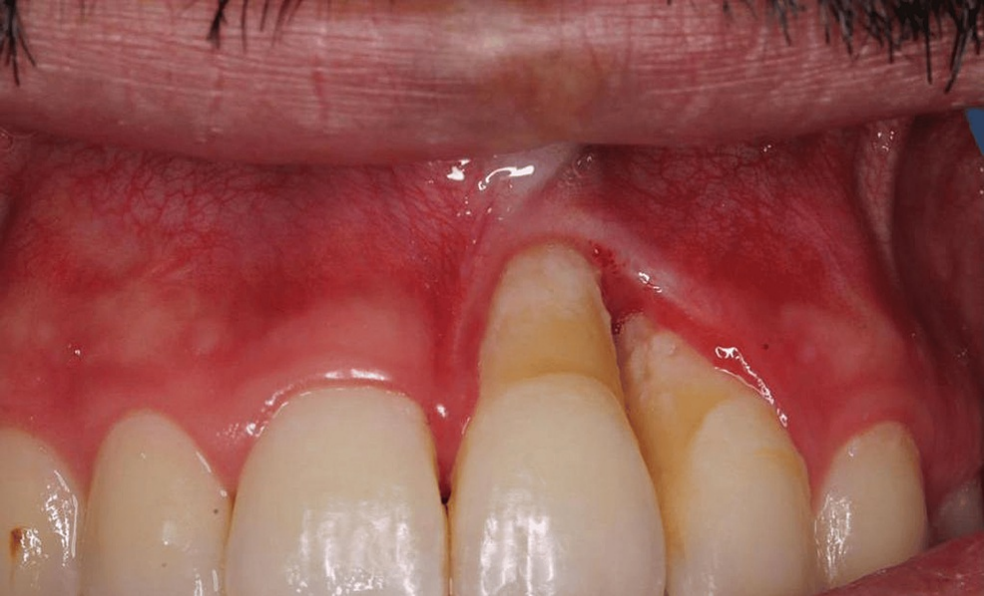
Intra-oral photo showing persistent recession and bone loss on the involved teeth

**Figure 7:**
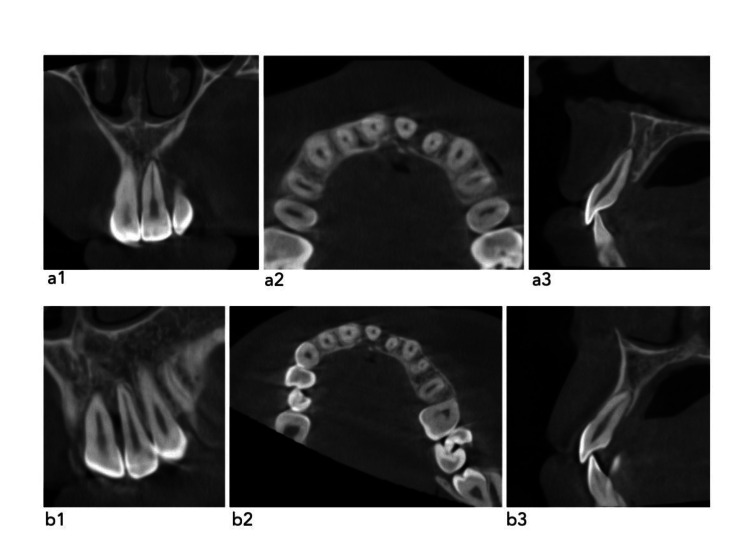
CBCT view a1. Corrected coronal image of tooth #21, a2. Corrected axial image of tooth #21, a3. Corrected sagittal image of tooth #21 b1. Corrected coronal image of tooth #22, b2. Corrected axial image of tooth #22, b3. Corrected sagittal image of tooth #22 CBCT: cone beam computed tomography

**Figure 8:**
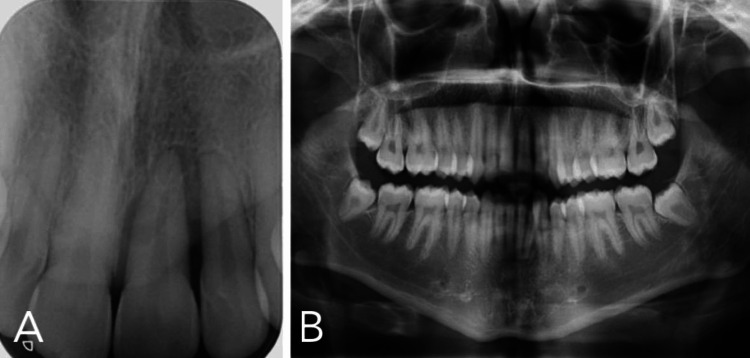
Panoramic radiograph and periapical radiograph of the upper anteriors A. Periapical radiograph of the upper anteriors showing vertical bone loss on #21 and #22 and widening of the periodontal ligament space with loss of lamina dura; B. Panoramic radiograph taken on the same day showed sound dentition with no marginal alveolar bone loss detected on the first molars

The patient underwent treatments in collaboration with his dermatologist that included immunosuppressants, 15 mg once per week of Methotrexate 2.5 mg (Methotrexate SPC ®), and Elidel (pimecrolimus MEDA, Germany ®) cream (1%) applied twice daily. The topical corticosteroids included Kourzeq (triamcinolone acetonide, USP ®) paste 0.1% applied directly to the lips and Dermovate (Clobetasol propionate GSK, Saudi Arabia ®) cream 0.05% applied intra-orally twice per day. Furthermore, topical medicine has been used in conjunction with excimer laser therapy started at a dose of 50 mJ/cm^2^, increased by 10 mJ/cm^2^, at each application, twice a week. Over two months, following the treatment plan, the patient's white patch demonstrated significant progress (Figure 5). Due to difficulty with commitment, the patient stopped using pimecrolimus cream, topical corticosteroids, and excimer laser on his own. He continued with Methotrexate (15 mg weekly) only for three more months. The next follow-up appointment was after six months, teeth #21 (the central) and #22 (the lateral) showed continuous progression in terms of bone resorption and recession (Figure 6), resulting in a less favorable periodontal prognosis (Figure 7, Figure 8). The patient has fair oral hygiene, so oral hygiene instructions were provided and scaling was done. Additionally, after consultation with his periodontist, the suggested treatment plan was orthodontic extrusion to reposition the gingival margin, extraction of teeth #21 and #22, bone graft, and implant placement.

## Discussion

The presented case of morphea underscores the complex and multifaceted nature of this rare autoimmune skin disorder [9]. Morphea is characterized by localized areas of skin hardening and thickening, typically presenting as oval or linear-shaped lesions with variable coloration, ranging from white to reddish-brown or purple. The exact etiology of morphea remains poorly understood, but it is thought to result from a combination of genetic, immunological, and environmental factors [10].

After examining more cases documented in the existing literature, it is evident that linear morphea affecting the oral cavity generally includes the mucosal lip and upper front teeth, as observed in our case [11-13]. The extent of gingival recession, alveolar bone loss, tongue atrophy, and tooth mobility varied among patients, and it was related to how far the disease had progressed when first diagnosed [11-13].

The clinical features in our case align with the classic presentation of morphea, demonstrating the diagnostic challenge it poses [9]. The diagnosis is mainly clinical, relying on the characteristic features of the lesions and patient history [10]. This aligns with the recommendation that a histopathological examination be considered in cases with atypical clinical features or when doubt exists about the diagnosis.

The complexity of differential diagnosis is due to the complicated clinical presentation combined with the asymptomatic nature of the lesion, adding to the complexity of the decision-making. Morphea commonly presents as pigmented mucosal lesions, described as scar-like or as an indurated whitish lesion [14]. These clinical features may bear a resemblance to other persistent white patches on the oral mucosa characterized by a plaque-like appearance such as plaque-like lichen planus, leukoplakia, chronic hyperplastic candidiasis, and oral submucous fibrosis. Some of these conditions are recognized as premalignant lesions, adding to the importance of a clinical assessment [15]. Lichen sclerosus et atrophicus (LSA) is an infrequently encountered disorder of the oral cavity that usually presents clinically as a distinct white patch of the oral mucosa-associated gingival recession, a widening of the periodontal space, a loss of periodontal attachment, and potentially as tooth loss like our case. The microscopic features of LSA are sufficiently characteristic to enable its distinction from localized scleroderma. However, in rare cases, overlapping histopathological features do exist. There is epithelial hyperplasia with hyperkeratosis, focal hydropic degeneration of the basal cells, subepithelial hyalinization, and a band-like lymphocytic infiltrate below the hyalinized area. The subepithelial elastic fibers are reduced in LSA [16-19]. Unlike the typical presentation, our patient did not exhibit a band-like infiltrate; instead, a noticeable perivascular lymphocytic infiltrate was apparent. For this reason, the diagnosis of localized scleroderma was rendered.

The investigation into the malignant transformation of white lesions has been a major topic of research in recent years. These studies have systematically identified and classified white lesions as premalignant lesions, providing insight into their role as early indicators of progression toward malignancy. For example, leukoplakia is a white lesion that appears in the oral mucosa. Traditionally, leukoplakia has been classified clinically as homogeneous or non-homogeneous. In a homogeneous lesion, the lesion appears as a uniformly white lesion with a flat (or slightly wrinkled) surface. In contrast, in a non-homogeneous lesion, it manifests as a combination of white or red hues (commonly called “erythroleukoplakia”) with a flat, speckled, or nodular surface [20].

The WHO’s 1978 definition delineates leukoplakia as a term employed to identify white plaques that do not appear to be caused by any other known disease or disorder and that may or may not carry an increased risk of cancer. Moreover, leukoplakia is a clinical term, and the histology of this lesion is non-specific, showcasing potential features such as atrophy or hyperplasia (acanthosis). It also might or might not exhibit epithelial dysplasia [21].

In a systematic review and meta-analysis of the malignant transformation rate by subtype, the clinical rate of leukoplakia is underscored. The analysis reveals that 9.5% of these lesions undergo a malignant transformation at a rate of 1.56% per year. The significant risk of malignant progression requires strict surveillance measures and the implementation of appropriate management strategies [15].

Additionally, lichen planus is an oral potentially malignant disorder. Lichen planus is a chronic inflammatory disease that exhibits several immune pathologies. It is a cell-mediated immune disease of an unknown cause [21]. Mainly occurring in middle-aged persons, the incidence is higher in women. The defining characteristic of oral lichen planus is that it appears as white papules that enlarge and coalesce to form a reticular, annular, or plaque pattern in the presence or absence of atrophy or erosion [22]. In the same systematic review previously referenced, lichen planus has a low risk of overall malignant transformation at only 1.4%, undergoing a transformation rate of 0.28% annually [15]. Furthermore, another systematic review and meta-analysis comprised 82 studies, and 26,742 patients reported a 1.14% probability of undergoing malignant transformation for oral lichen planus. These results highlight the significance of appropriate follow-up plans for all lichen planus patients to identify any changes in lesions that might indicate a potential cancerous transformation [23].

Additional differential diagnoses that may exhibit similarities and clinical resemblance in presentation such as chronic hyperplastic candidiasis, which is a prototypical oral lesion resulting from chronic Candida infection. characterized by its typical presentation in the anterior buccal mucosa bilaterally with diffuse margins [24,25]. There may also be frictional keratosis induced by mechanical irritation. Clinically, the lesions may appear as indistinct areas of grayish or whitish papules and plaques. Peeling and shredded keratin could be seen in the affected area, giving the area a macerated look [26]. Oral submucous fibrosis is a chronic progressive scarring oral condition exhibiting a burning sensation in the mouth, stiffness of the oral mucosa, ulceration, and blanching of the mucosa, as well as a gradual restriction of the mouth opening [25-27]. Oral submucous fibrosis is also known to have a significant rate of malignant transformation of 5.2% and a yearly evolution rate of 0.98% to cancer. In the clinical diagnosis and therapy of such lesions, taking into account this non-negligible risk of progression to cancer is essential [15]. Lastly, the snuff dipper’s pouch usually results from the direct impact of smokeless tobacco on the oral mucosa, with a wrinkled or fissured surface [25].

In the initial stages, both the symptoms and clinical signs, as well as the histological presentation, may lack distinctive characteristics [28] and exhibit subtle histologic features that may resemble those observed in psoriasis or lichen planus [28]. However, as the lesions mature, a distinct absence of elastic fibers is evident, without the presence of a band of lymphocytes at the epithelium connective tissue junction, distinguishing it from lichen planus [29].

The treatment of morphea is tailored to the patient's individual presentation and may include topical corticosteroids, phototherapy, and systemic immunosuppressive agents [30].

Recent research in morphea is shifting toward patient-centered care, as reflected in this case. Patient-reported outcomes, such as quality of life, pain, and psychological well-being, are now recognized as pivotal endpoints in clinical trials [31]. The patient’s perspective and experience of the disease are gaining importance in terms of guiding treatment decisions and improving overall patient care.

Additionally, genetics and biomarker research offers promise for the future [9]. Recent studies have identified genetic susceptibility factor associations with morphea and found with the HLA class II allele DRB1*04:04 and class I allele HLA-B*37 [2,10]. The possible presence of autoantibodies, such as antinuclear antibodies (ANA), single-stranded DNA (SS DNA), and antihistone antibodies as biomarkers, supports a role for autoimmune dysregulation [2,10], which may aid in early diagnosis and prediction of disease progression. This suggests a potential avenue for more targeted and effective treatments in the future.

Limitations

Multiple treatment modalities were effective in this case but the patient faced difficulty in following the treatment plan as a result of the combination of multiple treatments. This is why Methotrexate was the only treatment chosen for the patient to continue with. Using multiple medications can make it challenging to identify which one is delivering the desired effect but in this case, the dermatologist discontinued the other medications and relied exclusively on Methotrexate, as it has appeared effective and a safe treatment option for morphea patients [32], making it the most likely treatment responsible for the outcome.

## Conclusions

This case of morphea underscores the complexity of its diagnosis and management. While the clinical features are the primary diagnostic tool, a histopathological examination may be necessary in atypical cases. Treatment options may vary, and the response can be unpredictable. A multidisciplinary approach to care, including the involvement of rheumatologists and physical therapists, is essential. This case highlights the evolving understanding of morphea with an increasing focus on patient-reported outcomes and promising genetic and biomarker research, which may help prepare for more effective, personalized treatments in the future.
